# Epidemiological characteristics of three herpesviruses infections in children in Nanjing, China, from 2018 to 2023

**DOI:** 10.3389/fcimb.2024.1448533

**Published:** 2024-10-03

**Authors:** Mingwei Wei, Yang Zhang, Zhibin Li, Qi Liang, Tong Cao, Jingjing Ma

**Affiliations:** ^1^ NHC Key Laboratory of Enteric Pathogenic Microbiology, Jiangsu Provincial Center for Disease Control and Prevention, Nanjing, Jiangsu, China; ^2^ Department of Clinical Laboratory, Children’s Hospital of Nanjing Medical University, Nanjing, China; ^3^ Department of Immunology, Nanjing Medical University, Nanjing, China

**Keywords:** herpesviruses, HSV-2 (herpes simplex virus type 2), Epstein-Barr virus, Cytomegalovirus, children

## Abstract

**Objective:**

To evaluate the epidemiology characteristics of Herpes simplex virus type 2 (HSV-2), Epstein-Barr virus (EBV) and Cytomegalovirus (CMV) infection in children from January 2018 to December 2023, in Nanjing, China.

**Methods:**

We conducted a retrospective analysis of 21,210, 49,494 and 32,457 outpatients and inpatients aged 1 day to 17 years who were subjected to the three herpesviruses (HSV-2, EBV, and CMV) nucleic acid testing from January 2018 to December 2023, respectively. Demographic information, laboratory findings, etc. were collected and analyzed. HSV-2, EBV and CMV nucleic acid testing were performed by real-time PCR.

**Results:**

The total rate of detection of the three herpesviruses for all specimens was 0.32% (67/21,210), 14.99% (7419/49,494), and 8.88% (2881/32,457), respectively. A declining trend in the incidence of viral infections over the years was observed for the three herpesviruses (all *P*<0.05). The detection rate for HSV-2, EBV, and CMV was highest among patients aged 1-3 years, 3-7 years, and 28 days to 1 year, respectively (all *P*<0.05). The presence of HSV-2 and CMV infection did not exhibit a discernible seasonal pattern, whereas EBV typically demonstrated an elevation during the summer and autumn.

**Conclusion:**

EBV and CMV were both prevalent among children in China, except for HSV-2. The annual prevalence of the three herpesviruses show decreasing trend from 2018 to 2023, and no difference in gender (except for EBV). EBV infections usually occur in the summer and autumn, whereas HSV-2 and CMV do not exhibit significant seasonality. The positivity rate of HSV-2 is highest in 1-3 years, EBV is highest in 3-7 years, and that of CMV is highest in 28 days to 1 year. Positive detection rates are higher in outpatients than in inpatients.

## Introduction

Human herpesviruses (HHV1-8) are ubiquitous human pathogens belonging to the *Herpesviridae*, a large family of double-stranded DNA viruses, which is divided into three subfamilies: alphaherpesviruses [varicella-zoster virus (VZV), herpes simplex virus type 1 and 2 (HSV-1 and HSV-2)], betaherpesviruses [Human herpesviruses 6 (HHV-6), HHV-7 and human cytomegalovirus (HCMV)], and gammaherpesviruses [Epstein-Barr virus (EBV) and Kaposi’s sarcoma-associated herpesvirus (KSHV)] (Lan and Luo, 2017; Olsson et al., 2017; Noor et al., 2018). Moreover, herpes virus infections are life-long, enabling them to establish latency after primary infection prior to reactivation later in life.

HHVs are widely distributed worldwide, and more than 90% of the human population is infected by one or multiple HHVs (Lan and Luo, 2017), which can cause multiple diseases, such as Herpes simplex virus encephalitis (HSE) (Armangue et al., 2018; Piret and Boivin, 2020), Newborn Hearing Loss (Kadambari et al., 2020), genital herpes, infectious mononucleosis (IM) (Kuri et al., 2020; Sharifipour and Rad, 2020; Fedyanina et al., 2021), etc. HSV (including type 1 and type 2), EBV, and CMV are commonly present herpes viruses in human beings and are typically acquired during childhood. The neonatal herpes simplex virus (HSV) infection is an uncommon but severe disease with a high case-fatality rate (CFR) caused by HSV acquired during the neonatal period (Lao et al., 2019). It has been proven that primary or reactive EBV infection is associated with a variety of diseases, such as infectious mononucleosis (IM), respiratory infections, encephalitis, malignant lymphoma, nasopharyngeal carcinoma, aplastic anemia, hemophagocytic lymphohistiocytosis (HLH), immune dysfunction, and autoimmune diseases (Kasahara and Yachie, 2002; Shi et al., 2020; Shi et al., 2021; Ye et al., 2023). CMV is the most common viral cause of congenital infection worldwide and is the leading nongenetic cause of sensorineural hearing loss in children (Lan and Luo, 2017; Liu et al., 2017). However, all of three viruses’ primary infections in childhood are often asymptomatic or atypical, which may lead to misdiagnosis or missed diagnosis. In addition, limited research has been conducted on the etiological epidemiology of the three herpesviruses in children, especially for HSV-2.

Hence, understanding the epidemiology and other factors related to the three herpesviruses in children is crucial to updating existing guidelines, promoting antenatal hygiene behaviors, enhancing the utility of neonatal screening, and facilitating the development of related vaccines. In this retrospective study, we applied quantitative real‐time polymerase chain reaction (qRT-PCR) assay to detect HSV-2, EBV, and CMV DNA in a substantial number of samples from children and adolescents at Children’s Hospital of Nanjing Medical University. Additionally, we further elucidated the epidemiological characteristics of HSV-2, EBV, and CMV among children in China, including age distribution, gender distribution, and seasonal patterns.

## Materials and methods

### Ethics statement

The study was approved by the Medical Ethics Committee of Children’s Hospital, Nanjing University School of Medicine (No. 202403007-1). Written consent was waived due to the use of pre-existing routine medical data for this retrospective research, and did not require additional biological samples.

### Study population and sample collections

The study participants included outpatients and inpatients aged 0-17 years admitted in the Children’s Hospital of Nanjing Medical University and subjected to HSV-2, EBV, and CMV nucleic acid testing from January 2018 to December 2023. The data were obtained from outpatients and inpatients in the electronic medical record system, and the data analysis was anonymous. Demographic data and laboratory tests were recorded for each participant. All experiments conducted in this study adhered to the applicable guidelines and regulations. We collected samples of EDTA whole blood, plasma, cerebrospinal fluid, alveolar lavage fluid, sputum, urine, and secretions to detect CMV. For EBV detection, we used samples of EDTA whole blood, plasma, cerebrospinal fluid, alveolar lavage fluid, and sputum. We utilized samples of EDTA whole blood, plasma, cerebrospinal fluid, and secretions for HSV-2 detection.

### Specimens nucleic acid testing

The nucleic acids of all samples were extracted using Xi’an Tianlong nucleic acid extraction kit (Tianlong Technology). Subsequently, real-time quantitative fluorescence PCR was performed to detect the content of HSV-2/EBV/CMV in the samples by SLAN96P (Shanghai Hongshi Medical Technology). Detection Kit (Real-Time PCR) produced by Daan Gene, Guangzhou, China, was used in this study. HSV-2/EBV/CMV nucleic acid load detection was performed according to the protocol of the detection kits, and a negative, critical, positive, and four quantitative standards were used in each test.

### Statistical analysis

Statistical analysis was performed using the Statistical Package for Social Sciences (SPSS; version 24.0). The chi-square test was used to compare the classified variable groups, and the cartogram was drawn using Excel software. All reported P values were two sided, and *P*<0.05 was considered as statistically significant.

## Results

### Demographic characteristics

From January 2018 to December 2023, a total of 21,210, 49,494, and 32,457 eligible specimens were collected for Herpes simplex virus type 2 (HSV-2), Epstein-Barr virus (EBV), and Cytomegalovirus (CMV), respectively. In total, 1349, 16,750, and 3848 specimens were from outpatients, while 19,861, 32,744, and 28,609 specimens were from inpatients among the HSV-2, EBV, and CMV groups. The ratio (male/female) of the three herpesviruses (HSV-2, EBV, and CMV) groups was 1.41, 1.44 and 1.40, respectively. The median patient age was 0.17, 4.00, and 0.58 years (all range 0–17 years) in the three herpesviruses groups, respectively. Among the HSV-2, EBV, and CMV groups, the seasonal distribution of patients sampled was 5268, 11,581, and 8188 in spring (March to May), 5564, 14,442 and 8510 in summer (June to August), 5123, 12,280 and 8089 in autumn (September to November), and 5255, 11,191 and 7670 in winter (December to February), respectively.

### Viral prevalence

The total detection rate of the three herpesviruses (HSV-2, EBV, and CMV) for all samples was 0.32% (67/21,210), 14.99% (7419/42,075), and 8.88% (2881/29,576), respectively (**Table 1**). The annual positive rates from 2018 to 2023 were 0.45%, 0.81%, 0.27%, 0.06%, 0.03% and 0.00% for HSV-2, 17.94%, 20.15%, 15.24%, 12.61%, 9.64% and 13.55% for EBV, and 12.11%, 11.34%, 9.95%, 8.61%, 5.60% and 4.96% for CMV. A statistically significant difference was found among six years (all *P*<0.05). A declining trend in the incidence of viral infections over the years was observed for the three herpes viruses (all *P*<0.05) (**Table 1**).

**Table 1:** Detection rates of the three herpesvirus (HSV-2, EBV, and CMV) in children from 2018 to 2023[n (%)].

Viruses infected	2018	2019	2020	2021	2022	2023	Total
HSV-2	4209(0.45%)	4429(0.81%)	3395(0.27%)	3359(0.06%)	2860(0.03%)	2958(0.00%)	21,210(0.32%)
EBV	7791(17.94%)	9231(20.15%)	7486(15.24%)	7992(12.61%)	7431(9.64%)	9563(13.55%)	49,494(14.99%)
CMV	5500(12.11%)	5805(11.34%)	5256(9.95%)	5844(8.61%)	5093(5.60%)	4959(4.96%)	32,457(8.88%)

**Figure 1:**
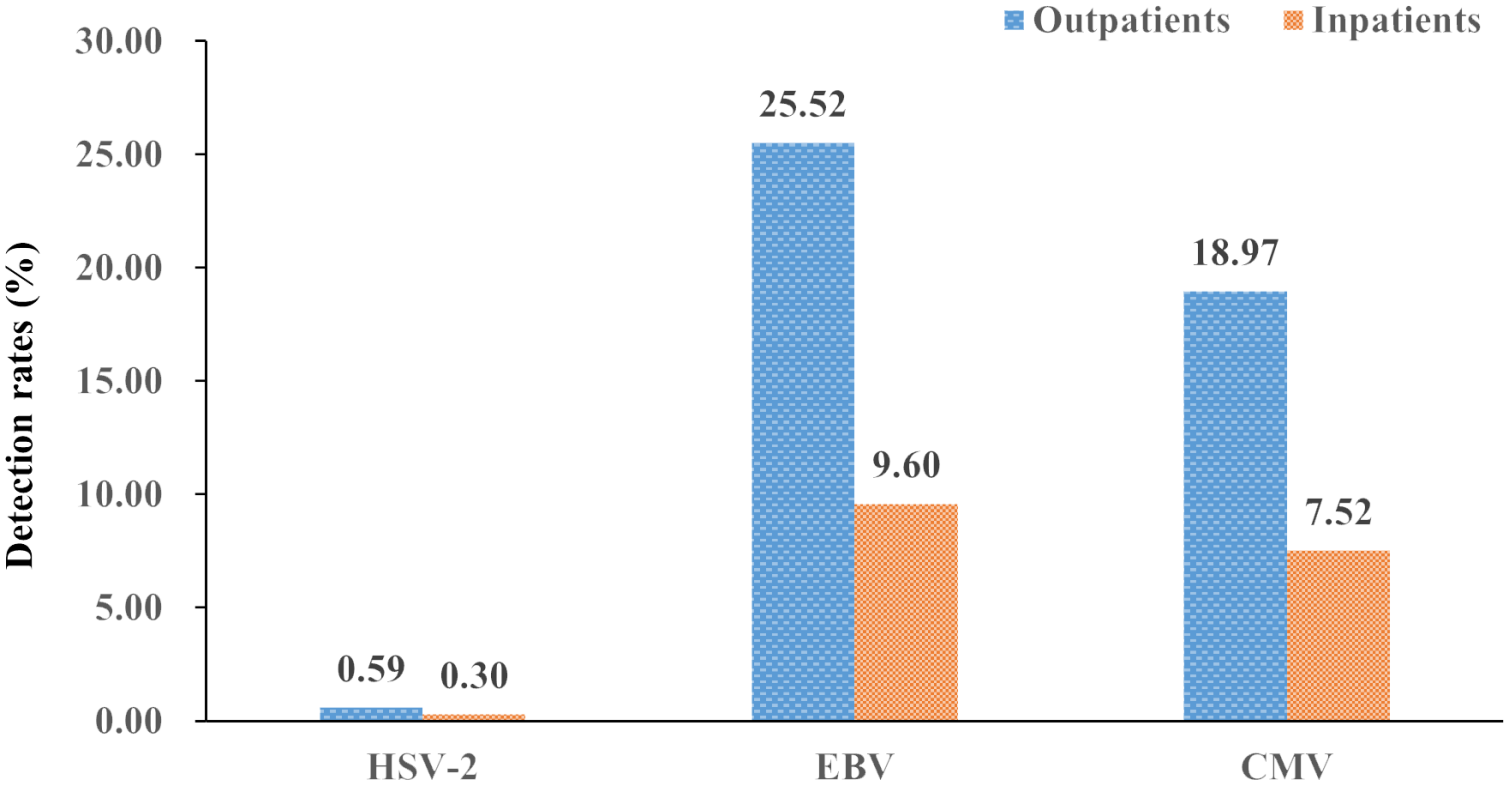
Detection rates of the three herpesviruses (HSV-2, EBV, and CMV) in outpatients and inpatients (%).

**Figure 2:**
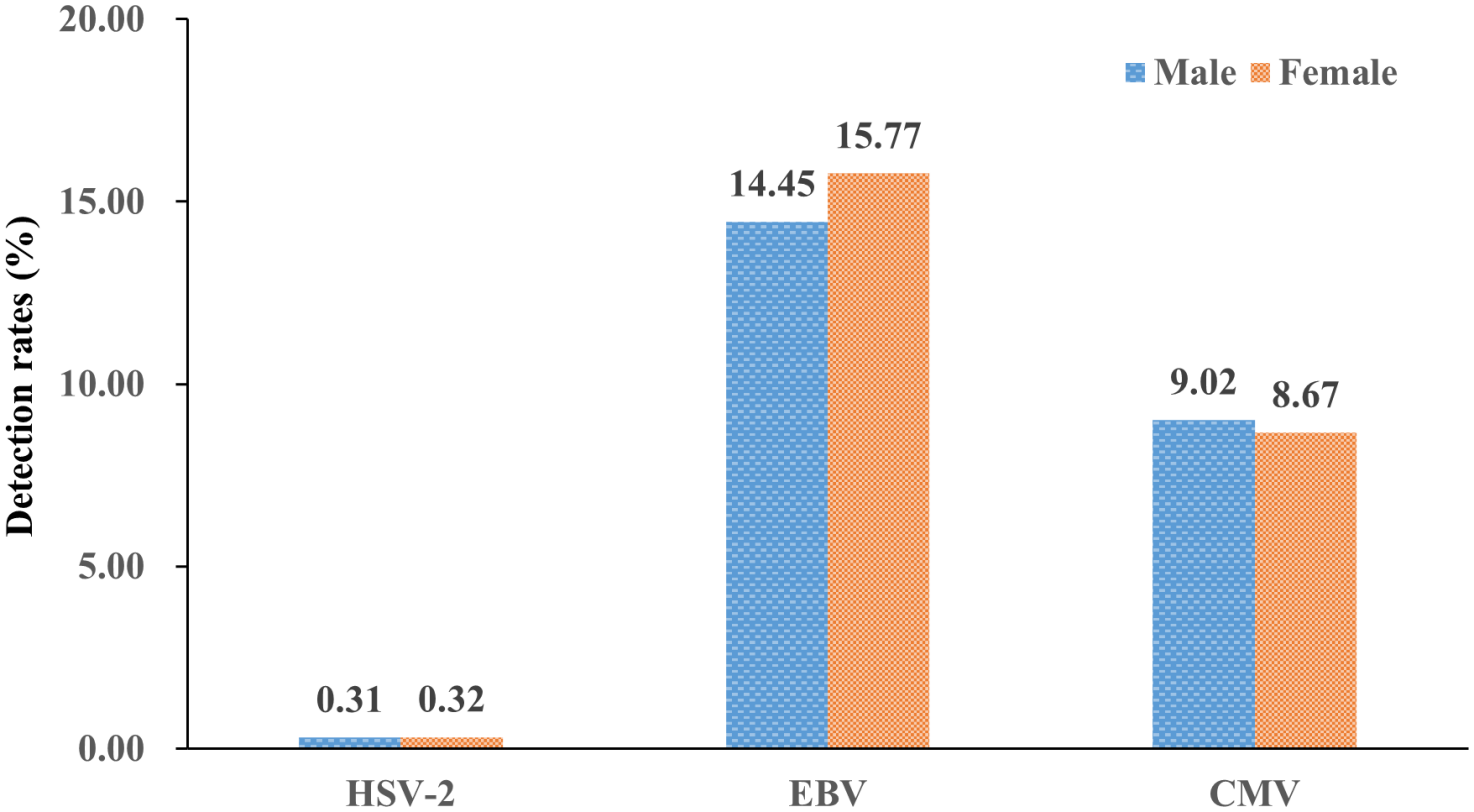
Detection rates of the three herpesviruses (HSV-2, EBV, and CMV) in male and female patients (%).

The total viral detection rates (all six years) for all outpatients were 25.52% and 18.97% in EBV and CMV, respectively, which was higher than that of inpatients (9.60% and 7.52%); the difference in detection rates was statistically significant for EBV and CMV (both *P*<0.05), except for HSV-2 (*P*>0.05) (**Figure 1**). The total viral detection rates (all six years, 2018-2023) of the HSV-2 and CMV were basically equal between males and females, and the differences were not statistically significant (all *P*>0.05), except for EBV (*P*<0.05) (**Figure 2**).

### Age distribution

**Figure 3:**
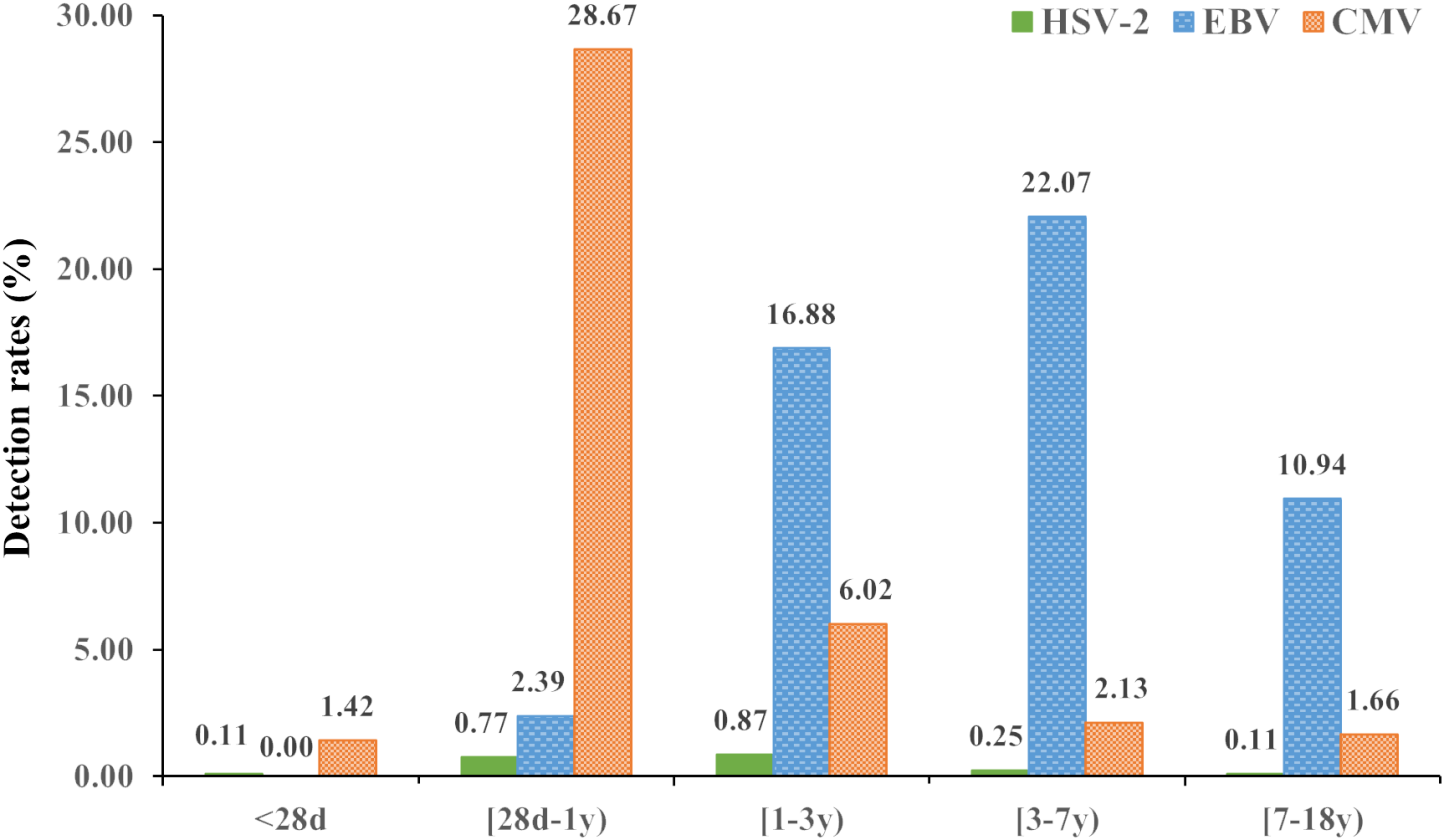
Detection rates of the three herpesviruses (HSV-2, EBV, and CMV) in different age groups (%).

The detection rates of three herpesviruses varied significantly among the five age groups, and the difference was statistically significant (all *P*<0.05) (**Table 2**). The detection rate for HSV-2, EBV, and CMV was highest among patients aged 1-3 years, 3-7 years, and 28 days to 1 year, respectively (all *P*<0.05) (Table2 and **Figure 3**).

**Table 2:** Detection rates of the three herpesvirus (HSV-2, EBV, and CMV) from different age groups [n (%)].

Age groups	HSV-2(n=21,210)		EBV(n=49,494)		CMV(n=32,457)	
	Positive (n=67)	Negative (n=21,143)	Positive (n=7419)	Negative (n=42,075)	Positive (n=2881)	Negative (n=29,576)
<28d	10(0.11%)	9208(99.89%)	0(0.00%)	73(100.00%)	134(1.42%)	9297(98.58%)
[28d-1y)	25(0.77%)	3240(99.23%)	158(2.39%)	6449(97.61%)	2326(28.67%)	5786(71.33%)
[1-3y)	21(0.87%)	2388(99.13%)	2130(16.88%)	10,485(83.12%)	210(6.02%)	3276(93.98%)
[3-7y)	7(0.25%)	2760(99.75%)	3623(22.07%)	12,791(77.93%)	96(2.13%)	4420(97.87%)
[7-18y)	4(0.11%)	3547(99.89%)	1508(10.94%)	12,277(89.06%)	115(1.66%)	6797(98.34%)
χ^2^	0.34		126.05		368.23	
*P* value	0.56		<0.01		<0.01	

### Seasonal distribution

**Figure 4:**
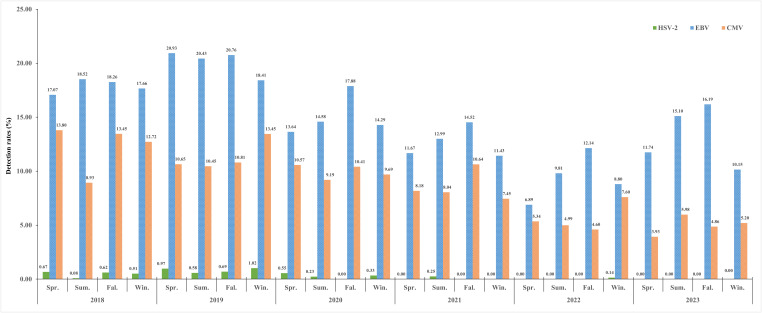
Seasonal distribution of the three herpesviruses (HSV-2, EBV, and CMV) from 2018 to 2023.

The overall viral detection rates (all six years) of the three herpesviruses in spring, summer, autumn, and winter were 0.42%, 0.22%, 0.25% and 0.38% for HSV-2, 13.96%, 15.50%, 16.63% and 13.59% for EBV, and 8.73%, 7.99%, 9.35% and 9.52% for CMV, respectively. A histogram for six study years showing the seasonality distribution of the three herpesviruses pathogens was drawn, and the detection rates decreased year by year (all *P*<0.05) (**Figure 4**). Overall, we did not observe a distinct seasonality profile in HSV-2 and CMV infection; the rate of HSV-2 infection was extremely low throughout the year, whereas the CMV infection remains consistently high. EBV infection exhibited a distinct seasonal profile and was detected more often during the summer (June and August) and autumn (October-November) (**Figure 4**).

## Discussion

This is the first retrospective study to investigate the epidemiology of the three herpesviruses, HSV-2, EBV, and CMV, in inpatient and outpatient children in Nanjing, China. Our findings provide a preliminary epidemiological profile and trends of three herpesviruses pathogens in inpatient and outpatient children.

In this study, it was found that the overall rates of HSV-2, EBV, and CMV positivity were 0.32%, 14.99%, and 8.88%, respectively. These results are lower than the HSV-2 and CMV seroprevalence levels of 9.9%-63.4% (HSV-2) (Sanchez-Aleman et al., 2018; Reward et al., 2019; James et al., 2020; Ayoub et al., 2021) and 23.7%-98.1% (CMV) (Zuhair et al., 2019; Hoehl et al., 2020; Winter et al., 2020; Rico et al., 2021) observed in foreign, and 3.4%-15.3% for HSV-2 (Zhang et al., 2014; Lin et al., 2011; Huai et al., 2019) and 38.6%-98.1% for CMV (Huang et al., 2021; Zhou et al., 2021; Huang et al., 2022) were observed in China, respectively. However, the seropositive rates of EBV varied widely among studies (Beader et al., 2018; Cui et al., 2018; Franci et al., 2019; Tang et al., 2020), ranging from 8.9% to 98.0%. The reasons why HSV-2, EBV, and CMV in this study are so different from previous studies may be as follows: first, PCR was used for detection in this study, while serological methods were used in the above studies; second, each study came from different countries and regions were economic and sanitary conditions vary widely. Third, the age, type, and specimen of participants in different studies varied widely. We also found that the annual positive rates of HSV-2, EBV, and CMV showed a downward trend from 2018 to 2023 (all *P*<0.05). The positive rate reduced remarkably from 2020 to 2023 for HSV-2 and CMV, which also reduced largely from 2020 to 2022 and increased largely in 2023 for EBV. The possible reasons for the above phenomenon are mainly due to the COVID-19 pandemic, resulting in the cancellation of public gatherings, delayed opening of schools, enhanced personal prevention and control measures (such as frequently washing hands and wearing masks), strict traffic control, etc. In addition, during the epidemic, maybe some patients with mild symptoms choose not to seek medical treatment at a hospital, so the actual number of cases may be underestimated. These similar phenomena have also been reported in other studies (Ding et al., 2020; Xiao et al., 2021). Additionally, this study found that EBV detection focused on school-aged kids (median age: 4.00 years), while HSV-2 and CMV were more prevalent in preschoolers (median ages: 0.17 year, 0.58 year). Post-pandemic, EBV rates rosed likely due to increased socializing among school-aged children, whereas HSV-2 and CMV rates slightly declined, likely reflecting different exposure patterns in preschoolers.

In previous studies, we only focused on EBV prevalence in hospitalized children (Chen et al., 2013). In the present study, we first studied the epidemiology characteristics of the three herpesviruses (HSV-2, EBV, and CMV) in both outpatient and inpatient children. The positive rate of outpatients in EBV and CMV was higher than that in inpatients (both *P*<0.05), while the positive rate of HSV-2 in outpatients and inpatients was basically equal (*P*>0.05). In the aspect of gender, a similar positive rate of the three herpesviruses in females and males was observed (both *P*>0.05), except for EBV, which was slightly higher in females than in males (*P*<0.05).

We found that the positivity rate of HSV-2, EBV, and CMV infection varied among different age groups. The positive rate of HSV-2 in each age group was low but was relatively high in the 1-3 years group. The participants of the previous related studies were mainly concentrated on the above 14 years old, which could not be compared (Xing et al., 2016; Huai et al., 2019). The peak detection rate of EBV was shown in the 3-7 years group (22.07%). In a similar study conducted in Shiyan and Suzhou, China, the prevalence of EBV DNA-positive peaked in <4 years and 3-4 years, respectively (Devkota et al., 2018; Shi et al., 2022). The differences described above may be mainly due to sample type, geographic location, economic, and sanitary conditions. CMV showed a peak detection rate in 28 day-1 year group. A possible explanation for this pattern is that the transmission routes in children occur mainly via close contact with their mothers, and the immune system is immature, which was also confirmed in other studies (Zuhair et al., 2019; Lanzieri et al., 2021).

This study adds to understanding seasonal variations of the three herpes viruses infections in Nanjing, China. Through this study of the three viruses during the 6-year study period, we found that the infection rate for HSV-2 showed mainly sporadic and no evident seasonality, which may be caused by the route of its transmission (such as contact with herpetic lesions, mucosal surfaces, genital secretions, or oral secretions) (Cole, 2020). We found that the infection for EBV was relatively high in summer and autumn, which was similar to previous research (Shi et al., 2022) but inconsistent with the findings in Hangzhou and Suzhou, China (Liu et al., 2017; Ayoub et al., 2021). Besides, we also found that the infection for CMV showed no apparent seasonal variation, and this similar phenomenon had also been reported in previous studies (Zhang et al., 2017; Chen, 2019).

This study had some advantages and limitations. Advantages: (1) the sample size of this study is relatively large, and the three herpesviruses were continuously monitored for six years. (2) The PCR DNA detection was used in this study, which has higher sensitivity and higher specificity than serology techniques. (3) The epidemiological surveillance of the three herpesviruses was performed for the first time on minors in Nanjing, China. Limitations: (1) Up to the present, the HSV-1 PCR tests have not been approved for clinical practice by the National Medical Products Administration (NMPA) in China. Therefore, the detection of HSV in this study is only for HSV type 2. (2) The subjects of this study were outpatient and inpatient children in the hospital rather than the general population, which may lead to a high positive detection rate. (3) This study only analyzed the infection characteristics of children without analyzing their corresponding clinical characteristics. (4) Co-infection analysis was not conducted in this study. Despite the above shortcomings, this study provides preliminary evidence for the prevalence of HSV-2, EBV, and CMV among children in Nanjing, China.

## Conclusion

In conclusion, our study showed that the overall prevalence of HSV-2 was 0.32%, EBV 14.99%, and CMV 8.88% in outpatient and inpatient children, and the annual incidences of HSV-2, EVB, and CMV show a decreasing trend from 2018 to 2023. HSV-2 and CMV show no clear seasonal variation, whereas EBV typically increases during the summer and autumn. Outpatients were more often found to be positive detection for EBV and CMV but this was basically equal to inpatients for HSV-2. The peaked detection rate for HSV-2, EBV, and CMV is among patients aged 1-3 years, 3-7 years, and 28 days to 1 year, respectively.

## Data Availability

The raw data supporting the conclusions of this article will be made available by the authors, without undue reservation.
